# Depressive Symptoms in Older African Immigrants: A Descriptive Study

**DOI:** 10.1093/geroni/igaa057.1075

**Published:** 2020-12-16

**Authors:** Manka Nkimbeng, Zachary Baker, Janiece Taylor, Sarah Szanton, Tetyana Shippee, Joseph Gaugler

**Affiliations:** 1 Johns Hopkins University school of Nursing, Minneapolis, Minnesota, United States; 2 University of Minnesota, Minneapolis, Minnesota, United States; 3 Johns Hopkins School of Nursing, Baltimore, Maryland, United States; 4 Johns Hopkins University, Baltimore, Maryland, United States

## Abstract

In FY 2018-2019, the National Institutes of Health devoted $2,387,505,711 to projects studying depression. Before and following their arrival into the United States stressful life circumstances may render African immigrants particularly at risk for depression. The objective of this study is to provide an estimate and identify correlates of depressive symptoms in older (≥50 years) African immigrants. We performed secondary data analyses of the Older African Immigrant Health study (n = 148). Bivariate analyses evaluated associations between depressive symptoms and sociodemographic and immigration-related factors. Depressive symptoms were measured with the PHQ-8 scale and scores of ≥ 5 were considered indicative of depressive symptoms. The mean age of participants was 62 years (SD:8.2), 61% were female, 30% had less than high school education, and 58% reported having health insurance coverage. Thirty percent of the sample had depressive symptoms (PHQ-8 score of ≥ 5) but only one individual would be classified as having moderately severe or severe depression (PHQ-8 ≥15). Depressive symptoms did not differ by age, marital status, education, or income. There was a statistically significant difference in depressive symptoms by reason for migration, recruitment location, and employment status. Although only one participant would be classified as severely depressive, a large proportion of this sample had depressive symptoms. Mental health concerns were reported as a significant health problem for African immigrants visiting a community service organization in New York. More research is needed to examine the prevalence, immigration-related correlates, predictors, and health ramifications of depression in older African immigrants.

